# Prevalence and course of somatic symptoms in patients with stress-related exhaustion: does sex or age matter

**DOI:** 10.1186/1471-244X-14-118

**Published:** 2014-04-23

**Authors:** Kristina Glise, Gunnar Ahlborg, Ingibjörg H Jonsdottir

**Affiliations:** 1 The Institute of Stress Medicine, Sahlgrenska Academy at the University of Gothenburg, Carl Skottsbergs gata 22B, Göteborg SE-413 19, Sweden; 2 Occupational and Environmental Medicine at the Department of Public Health and Community Medicine, Sahlgrenska Academy at the University of Gothenburg, Gothenburg, Sweden

**Keywords:** Stress, Exhaustion, Burnout, Sex, Age, Somatic symptoms

## Abstract

**Background:**

Both mental and somatic symptoms are commonly reported in patients with stress-related problems. We have explored the prevalence of somatic symptoms in patients seeking medical care for stress-related mental health problems and followed the course of illnes alongside with that the patients receive multimodal treatment.

**Method:**

This study comprises data from 228 patients (69% women, mean age 43 years) who fulfilled the criteria for Exhaustion Disorder (ED). Somatic symptoms were assessed at baseline and after 3, 6, 12 and 18 months using the one-page questionnaire Primary Care Evaluation of Mental Disorders. Prevalence of different symptoms was compared between men and women and patients, over and below 40 years of age, and possible predictors of recovery were explored.

**Results:**

Tiredness and low energy are the core symptom reported by the patients. Almost all (98%) reported at least one somatic symptom and 45% reported six symptoms or more, which was similar for men and women. Nausea, gas or indigestion are the most common symptoms (67%) followed by headaches (65%) and dizziness (57%). The number of symptoms reported was significantly related to the severity of mental health problems. The only difference between the sexes was that “chest pain” and “pain or problems during sexual intercourse” were more common among males. Patients over forty more often reported “pain in arms, legs or joints, knees, hips” and this was also the only symptom that did not significantly decline during treatment. Neither sex, age, symptom duration before seeking medical care, education or any other predictor tested was shown to predict recovery in patients reporting six symptoms or more.

**Conclusion:**

A heavy burden of somatic symptoms was generally seen in most patients with stress-related exhaustion. Somatic symptoms are equally common in males and females and in younger and older patients. The somatic symptoms seem to be mostly stress-related since all symptoms, except musculoskeletal pain, reduce with individualised treatment designed for stress-related mental problems. This study brings to attention the complicated burden of both somatic and mental symptoms in patients with stress-related exhaustion, raising several clinical implications of interest to discuss.

## Background

It is well known that chronic stress exposure can result in clinical symptoms and complaints, often referred to as stress-related disorders [1,2]. The term “stress-related disorder” has not been clearly defined, but is most commonly used to describe mental health problems mainly caused by psychosocial stress, such as fatigue, burnout, exhaustion, depression or adjustment disorder. Studies using the burnout concept are numerous in the literature and it is commonly defined as a mental condition that has developed as a result of continuous stress exposure particularly related to psychosocial factors at work [3]. The theoretical basis behind the available self-report instruments constructed to assess burnout differs. Attempts have been made to adapt the burnout concept to be more usable in clinical practice. In the Netherlands, clinical burnout as diagnosis has been suggested, using the diagnostic criteria of neurasthenia and adding the component that the problem should be work-related [4,5]. We have also recently suggested a cut-off for clinical burnout when using the Shirom-Melamed Burnout questionnaire (SMBQ) [6]. The clinical diagnosis “Exhaustion disorder” (ED) has been proposed by the National Board of Health and Welfare in Sweden to be used in clinical practice. ED defines patients with exhaustion that has developed as a consequence of identifiable stressor(s) that have been present for at least six months. The symptoms of ED and burnout are closely related, and we have previously shown that the majority of patients fulfilling the diagnostic criteria for ED can also be described as burned-out [7,8]. We have also recently shown that patients with ED do report both work-related and non-work related stressors as a plausible cause of the stress-related exhaustion. This supports the use of clinical diagnosis that does not only consider work-related stress exposure [9].

Persons seeking medical care mainly for stress-related exhaustion often report co-morbid depression and/or anxiety and consequently their mental burden of illness is high [10-13]. The relationship between mental and somatic symptoms is well recognized both from clinical experience and from the literature [14,15] and thus we expect the occurrence of somatic symptoms to be high in these patients. Several previous studies have shown that the prevalence of somatic symptoms is higher in primary care patients with depressive disorder than in patients not reporting any mental disorder [14,15]. Having multiple somatic symptoms has been shown to be a strong predictor of mental health problems such as depression and anxiety [16]. Somatisation disorder, which is as a tendency to experience and communicate somatic distress rather than cognitive response to psychosocial stress and to seek medical help for it, this condition is also often combined with mental health problems [17].

A high prevalence of somatic symptoms in patients with stress-related exhaustion could also be expected due to the fact that a high level of perceived stress is known to be related to many different somatic symptoms such as headache [18,19], gastrointestinal problems [20,21], palpitation [22] and musculoskeletal and joint pain [23]. Physical illness is more common among subject with burnout than others [24] and several studies have shown that burnout in working populations is related to increased level of different somatic symptoms such as gastrointestinal and cardiovascular symptoms [25] and neck and back pain [25,26] as well as to the general level of somatic complaints [27]. Few studies are available studying prevalence of somatic symptoms in a clinical population of patients suffering from exhaustion/clinical burnout and we are not aware of any study that have followed the course of symptoms for a longer period of time. One interesting aspect when studying symptoms and the burden of illness is sex differences. Women consistently report higher prevalence of somatic symptoms, particularly musculoskeletal pain, compared to men [28,29], and mental disorders such as depression and anxiety are more prevalent among women [30-32]. Burnout, mainly studied in working populations has in many studies been seen to be more common among women compared to men [33,34], while other studies have not seen any difference in prevalence of burnout between women and men [35]. Interestingly, we recently showed in a clinical patient population diagnosed with exhaustion that men and women did not differ regarding the burden of mental symptoms, measured as burnout, depression and anxiety [8]. Thus, the sex difference commonly seen for the prevalence of mental health problems does not seem to be reflected in the burden of symptoms in a clinical patient population [8].

In this study, we wanted explore the burden of somatic symptoms in a clinical patient population with stress-related exhaustion, and to explore the prevalence of several different symptoms. We hypothesize that both female and male patients would report a substantial number of somatic symptoms. Kroenke and co-workers (1998) showed that the sex difference, with women generally reporting higher levels of symptoms, was independent of psychiatric co-morbidity, measured as depression and/or anxiety [29]. A similar finding was reported by Haug and co-workers showing that women report a higher mean number of somatic symptoms than men irrespective of depression or anxiety diagnosis [36]. Given this, one could expect higher burden of somatic symptoms in women compared to men with stress-related exhaustion. The primary aim of the present study was to study the prevalence of somatic symptoms in patients with stress-related exhaustion and to follow the course of symptoms during 18 months alongside with that the patient receive multimodal treatment (MMT). Another aim was to explore if female and male patients, as well as younger and older patients, differ regarding the prevalence of somatic symptoms and course of symptoms over time. Baseline data collected at the first visit regarding socio-demographic factors, co-morbidity, duration of symptoms before seeking medical care, and use of antidepressant medication, were also evaluated as possible predictors of recovery in terms of reduction in the number of symptoms during the 18-month follow-up.

## Methods

### Subjects

The present study comprises data from 228 patients, 156 (69%) women and 72 (31%) men. All had been referred from Primary Health Care or Occupational Health Service Centres between 2004 and 2010 to a specialist clinic in western Sweden exclusively treating patients with stress-related mental disorders. The referral criteria were stress-related exhaustion with no apparent somatic disorder or abuse that could explain the exhaustion, and a maximal duration of sick leave of six months. All patients were ambulatory at the time of the study and none had received in-patient care due to their illness. Seventy-eight percent was on full-time or part-time sick leave.

The mean age for the group was 43.3 years (SD 9.3; range 24–63), with no difference between women (43.7; SD 9.7) and men (42.3; SD 8.4). When analysing possible age-related differences among the patients, we split into younger (24–39 years; *n = 82*) and older (40–63 years; *n = 146*). Baseline characteristics for the patients are shown in Table 1.

**Table 1:** Baseline characteristics of patients with stress-related Exhaustion Disorder (ED)

	**N**	**Total n (%)**	**Women (n = 156) n (%)**	**Men (n = 72) n (%)**	**p-value**^**6**^
Marital status	228				
– Married		171 (75)	119 (76)	52 (72)	0.510
– Single or other		57 (25)	37 (24)	20 (28)	
Education^1^	219				
– higher		157 (72)	116 (76)	41 (62)	**0.039**
– lower		62 (28)	37 (24)	25 (36)	
Duration of symptoms^2^	219				
– <1 year		93 (43)	62 (42)	31 (44)	0.709
– ≥1 year		126 (58)	87 (58)	39 (56)	
Co-morbid depression^3^	228				
– Yes		175 (77)	117 (75)	58 (81)	0.356
– No		53 (23)	39 (25)	14 (19)	
Co-morbid anxiety^3^	228				
– Yes		185 (81)	121 (78)	64 (89)	**0.042**
– No		43 (19)	35 (22)	8 (11)	
SMBQ^4^	192				
– <4		12 (6)	8 (6)	4 (7)	0.936
– ≥4		180 (94)	122 (94)	58 (94)	
HAD depression^5^	214				
– <10		140 (65)	102 (69)	38 (59)	0.157
– ≥ 11		74 (35)	47 (32)	27 (42)	
HAD anxiety^5^	213				
– <10		81 (38)	56 (37)	25 (40)	0.747
– ≥ 11		132 (62)	94 (63)	38 (60)	

^1^Higher education is one year of college or more ^2^ Self-reported duration of symptoms before seeking medical care for exhaustion, ^3^Clinical diagnosis, ^4^Mean total score of the Shirom Melamed Burnout Questionnaire, ^5^Total score on the respective subscale of the Hospital Anxiety and Depression Scale.

^6^p-value: Pearson’s Chi Square test was used to compare women and men.

### Inclusion criteria

Only patients fulfilling the diagnostic criteria for Exhaustion Disorder (Table 2) were included in this study. Co-morbidity of depression and/or anxiety was allowed and screened for. Patients with somatic disorders, such as generalised pain, thyroid disease, vitamin B-12 deficiency or obesity, which might explain the exhaustion, were excluded from this analysis, along with data from patients with alcohol abuse or serious psychiatric diagnoses other than depression and anxiety. Blood samples were taken for the purpose of differential diagnosis.

**Table 2:** Diagnostic criteria for stress-related Exhaustion Disorder as proposed by the Swedish National Board of Health and Welfare

**Diagnostic criteria for exhaustion disorders**		
**A**	Physical and mental symptoms of exhaustion with a minimum of two weeks duration. The symptoms have developed in response to one or more identifiable stressors which have been present for at least 6 months.	
**B**	Markedly reduced mental energy, which is manifested by reduced initiative, lack of endurance, or increase of time needed for recovery after mental efforts.	
**C**	At least four of the following symptoms have been present most of the day, nearly every day, during the same 2-week period:	
	*1 Persistent complaints of impaired memory.*	
	*2 Markedly reduced capacity to tolerate demands or to work under time pressure.*	
	*3 Emotional instability or irritability.*	
	*4 Insomnia or hypersomnia.*	
	*5 Persistent complaints of physical weakness or fatigue.*	
	*6 Physical symptoms such as muscular pain, chest pain, palpitations, gastrointestinal problems, vertigo or increased sensitivity to sounds.*	
**D**	The symptoms cause clinically significant distress or impairment in social, occupational or other important areas of functioning.	
**E**	The symptoms are not due to the direct physiological effects of a substance (e.g. a drug of abuse, a medication) or a general medical condition (e.g. hypothyroidism, diabetes, infectious disease).	
**F**	The stress-related disorder does not meet the criteria for major depressive disorder, dysthymic disorder or generalized anxiety disorder.	

### Diagnostic procedures

Three senior physicians carried out a diagnostic procedure, obtaining an extended anamnesis and performing a physical examination. The diagnostic procedure for ED has been previously described in detail [8]. If the patient meet the criteria for major depressive disorder, dysthymic disorder or generalised anxiety disorder, these diagnoses are set first and ED is set as a co-morbid condition. The assessment of depression and anxiety was standardized by using the Primary Care Evaluation of Mental Disorders (PRIME-MD) instrument [37]. Before consulting the physician the patients filled in the one-page PRIME-MD patient questionnaire that covers questions on both somatic as well as mental symptoms. Positive responses were followed up by the physician in a structured interview based on the PRIME-MD manual, conforming with the DSM IV criteria [37], for diagnostic assessment of depression and anxiety disorder. General anxiety, unspecific anxiety and/or panic disorders were classified as any anxiety disorder. During both referral and diagnostic procedures, special attention was paid to diagnostic criteria of chronic fatigue syndrome [38], and fibromyalgia [39], which share many symptoms with ED. Patients who fulfil the criteria for these diagnoses were referred to other clinics and thus data from such patients were not included in this study.

All patients thus fulfilled the diagnostic criteria of ED at baseline. Nine percent were diagnosed with ED alone and 10% also fulfilled the criteria for depression as a co-morbid condition, 15% for any anxiety disorder as a co-morbid condition and 67% for both depression and any anxiety disorder as co-morbid with ED (Table 1).

The prevalence of co-morbid depression did not differ between women and men or between younger (73%) and older (79%) patients but men reported more co-morbid anxiety than women (Table 1), as did younger (89%) compared to older patients (77%) (p = 0.023).

### Procedures

Data analysed in this study is registry data from a patient registry that has been collected during several years at the specialist clinic. The data used in this study was collected during the first visit at the clinic and then at follow-ups after 3, 6, 12 and 18 months. During this period all patients received multimodal treatment (MMT). All patients receive similar treatment and the content of the MMT treatment has been described in more detail previously [8]. When entering the treatment programme, 28% *(n = 64)* of the patients were already on antidepressants (ADs) and as a large number of the patients were clinically judged to be in need of such medication as many as 60% *(n = 136)* were on antidepressants after the 3-month follow-up. At the 18-month follow-up 53% *(n = 121)* were still on antidepressants. Frequent communication with the Social Insurance Office and the employer regarding sick leave was maintained, aiming for the earliest possible return to work.

### Measurements

Bivariate analysis was performed for each of the following baseline variables as possible predictors of recovery: clinical depression (yes or no), any anxiety disorder (yes or no), combined co-morbidity (ED or ED and depression, ED and any anxiety or ED combined with depression and any anxiety), marital status (married or “single or other”), sick leave (no sick leave, part-time or full-time sick leave), self-reported physical activity (sedentary lifestyle, light or “moderate physical activity or moderate to vigorous physical training”), use of ADs (yes or no), symptom duration and level of education. The level of education was defined as high if the person had completed one year of college education or more. Symptom duration was measured by asking the patients to estimate for how many years, before seeking medical care they had experienced the symptoms that they were seeking care for. Symptom duration was dichotomised into less than one year or more than a year. All data mentioned above was collected before the first visit to the clinic, using a postal questionnaire that all patients entering the treatment programme at the clinic were asked to fill in before their first visit.

The questionnaires regarding somatic symptoms, burnout, depression and anxiety, described below, were filled in by the patients at baseline and then during follow-up at 3, 6, 12 and 18 months.

### Somatic symptoms

The PRIME MD short patient form includes 16 questions on commonly reported symptoms in primary care patient populations: stomach pain, back pain, pain in arms, legs or joints (knees and hips included), menstrual pain or problems, pain or other problems during sexual intercourse, headache, chest pain, dizziness, fainting spells, feeling your heart pound or race (i.e. palpitation), shortness of breath, constipation, loose bowels or diarrhoea, nausea, gas or indigestion, feeling tired or having low energy, trouble sleeping, and eating being out of control. The patients were asked to answer yes or no to each question depending on whether the symptom had occurred often during the past month. The patients were then asked to fill out the same form at each follow-up. The symptoms “feeling tired or having low energy”, “trouble sleeping” or “eating being out of control” are rather considered as measures of mental symptoms and were thus excluded in this analysis of somatic symptoms. Two of these symptoms are also considered as cardinal symptoms of these patients, as 98% reported that they felt tired or had low energy and 85% reported that they had trouble sleeping.

Menstrual symptoms were also excluded from the analysis since one of the main aims of this study was to compare males and females. The remaining 12 symptoms were selected to represent the somatic burden of disease in patients with ED.

### Burnout and symptoms of depression and anxiety

The Shirom-Melamed Burnout Questionnaire (SMBQ) was used to measure symptom of burnout [40]. A mean score above 3.75 on SMBQ total score has been used as a cut-off to define high burnout based on quartile splits [41], and Stenlund and co-workers reported the mean score of the total scale in patients with clinical burnout to be 5.7 for women and 5.6 for men [42]. In the present study, a total mean score of ≥ 4.0 was used as a cut-off for burnout as it was used in our previous paper of a similar patient population [8]. This score is close to the cut-off point of ≥4.4 that our research group has suggested as the cut-off to be used as an indication of clinical burnout [6]. Since ED and burnout are closely related conditions, we expected almost all patients to score above this cut-off. The widely used Hospital Anxiety and Depression scale (HAD) was employed to assess self-reported symptoms of depression and anxiety. This scale was originally developed for non-psychiatric clinics to detect states of depression and anxiety [43]. A sum score above 10 was used to indicate probable depression and anxiety, respectively. Cronbach’s alpha for the total SMBQ was found to be 0.91, for HAD subscale anxiety 0.82 and for HAD subscale depression 0.81.

### Statistical analysis

Pearson’s Chi-Square test was used to compare men and women regarding baseline characteristics, except for age where Student’s *t*-test was used. The prevalence of symptoms during each follow-up was compared between men and women and the two different age groups (24–39 and 40–63 years) respectively by using Pearson’s Chi-Square test.

Somatic symptoms were analysed in three ways 1) as mean number and standard deviation (SD) of symptoms at each time point during the follow-up, 2) as a dichotomous variable where a total of 6 symptoms or more was set as cut-off level indicating a heavy burden of somatic symptoms, and 3) as prevalence of each symptom at baseline and during follow-up.

The cut-off of 6 symptoms and more was chosen according to Kroenke and co-workers that showed in primary care patients that there was a powerful relationship between the number of somatic symptoms and the likelihood of a mental disorder. At this particular burden of somatic symptoms 6 or more, referred to as multiple somatic symptoms, the likelihood of anxiety or depressive disorder was more than half of the population [15,44].

When analysed as mean number of symptoms, the non-parametric Mann–Whitney Test was used to compare different groups (men and women, younger and older) as the data was not normally distributed. The Mann–Whitney Test was also used to compare mean somatic symptoms in patients scoring over and under the median cut (5.45) on SMBQ. The non-parametric Wilcoxon’s signed rank test was used to test for significant changes in mean somatic symptoms between the measurement points. Comparing the prevalence of somatic symptoms using the HAD subscales, the cut-off 11 or more was used for both.

When somatic symptoms were treated as a dichotomous variable, the non-parametric Cochran’s Q test was used for testing change over time in the proportion of patients who scored above the cut-off level of six symptoms or more. McNemar’s test was then used for group-wise comparisons between two measurement points.

Cox’s regression with constant time at risk was used in bivariate analyses to identify possible predictors of recovery measured as scoring <6 somatic symptoms, at the follow-ups after 6, 12 and 18 months among patients who reported more than six somatic symptoms at baseline. Results were expressed as a prevalence ratio (RR) with 95% confidence intervals. The bivariate analyses were performed for each of the following baseline variables: clinical depression, any anxiety disorder, combined co-morbidity, marital status, symptom duration, sick leave, level of education, physical activity and use of ADs.

The level of significance was set as p < 0.05. The statistical package SPSS Statistics 19 was used for all statistical analyses.

### Ethics

The study was approved by the regional ethical review board in Gothenburg, Sweden and conducted according to the 1964 Helsinki Declaration. Only patients who consented to the use of their clinical data for research purposes were included.

## Result

### Prevalence of somatic symptoms

The mean number of symptoms reported by the patients at baseline was 5.3 (SD 2.4). This did not differ significantly between women (mean 5.3; SD 2.4) and men (mean 5.3; SD 2.6) (p = 0.790) or between younger (mean 5.2; SD 2.5) and older patients (mean 5.3; SD 2.4) (p = 0.798). Four patients (2%) reported no somatic symptoms; 26 patients (11%) reported one or two symptoms, 96 patients (42%) reported three to five symptoms and 102 patients (45%) reported six symptoms or more. The proportion reporting six or more symptoms was similar among males (42%) and females (46%) as well as among younger and older patients (both 45%).

Among the 12 somatic symptoms analysed in this study, problems with gas or indigestion were the most common symptoms reported (67%), followed by headaches and dizziness (Table 3). There were no differences in the prevalence of the different somatic symptoms between males and females, except that chest pain and pain or problems during sexual intercourse were significantly more common among men (Table 3). The only difference seen between older and younger patients was that pain in the arms, legs or joints was more common in the older patients. There was also an indication that problems during sexual intercourse might be more common in younger than older patients, but this difference did not reach statistical significance (Table 4).

**Table 3:** Percentage of women and men with Exhaustion Disorder (ED) reporting on the PRIME-MD* symptoms checklist at the first visit to the stress clinic that they have frequently experienced the respective symptom during the past month

**Symptom**	**Total group**	**Women**	**Men**	**p-value**^**1**^
	**(N = 228) %**	**(n = 156)**	**(n = 72)**	
	**(n)**	**% (n)**	**% (n)**	
Nausea, gas or indigestion	67 (153)	69 (108)	63 (45)	0.315
Headaches	65 (149)	64 (99)	69 (50)	0.378
Dizziness	57 (129)	l60 (94)	49 (35)	0.099
Constipation, loose bowels, or diarrhoea	54 (123)	54 (84)	54 (39)	0.964
Feeling heart pound or race	54 (122)	57 (89)	46 (33)	0.114
Back pain	51 (116)	49 (76)	56 (40)	0.337
Chest pain	47 (106)	42 (65)	57 (41)	**0.032**
Pain in arms, legs or joints, knees, hips	49 (111)	52 (81)	42 (30)	0.150
Stomach pain	45 (103)	47 (74)	40 (29)	0.313
Shortness of breath	23 (52)	22 (34)	25 (18)	0.592
Pain or problems during sexual intercourse	14 (31)	10 (16)	21 (15)	**0.030**
Fainting spells	4 (8)	3 (5)	4 (3)	0.714

*PRIME-MD = Primary Care Evaluation of Mental Disorders.

^1^p-value: Pearson’s Chi-Square test was used to compare the prevalence of the single somatic symptoms between women and men. Bold numbers indicate that the difference between the sexes is statistically significant (p < 0.05).

**Table 4:** Percentage of younger (24–39 years) and older (40–63 years) patients with Exhaustion Disorder (ED) reporting on the PRIME-MD* symptoms checklist at the first visit to the stress clinic that they have frequently experienced the respective symptom during the past month

**Symptom**	**Total group**	**Younger**^**1**^	**Older**^**2**^	**p-value**^**3**^
	**(N = 228)**	**(n = 82)**	**(n = 146)**	
	**% (n)**	**% (n)**	**% (n)**	
Nausea, gas or indigestion	67 (153)	62 (51)	70 (102)	0.237
Headaches	65 (149)	72 (59)	62 (90)	0.117
Dizziness	57 (129)	49 (40)	61 (89)	0.075
Constipation, loose bowels, or diarrhea	54 (123)	54 (44)	54 (79)	0.948
Feeling heart pound or race	54 (122)	49 (40)	56 (82)	0.283
Back pain	51 (116)	52 (43)	50 (73)	0.724
Chest pain	47 (106)	51 (42)	44 (64)	0.283
Pain in arms, legs or joints, knees, hips	49 (111)	33 (27)	58 (84)	**<0.001**
Stomach pain	45 (103)	48 (39)	44 (69)	0.558
Shortness of breath	23 (52)	24 (20)	22 (32)	0.669
Pain or problems during sexual intercourse	14 (31)	20 (16)	10 (15)	0.051
Fainting spells	4 (8)	5 (4)	3 (4)	0.400

^1^Younger: 24–39 years.

^2^Older: 40–63 years.

^3^p-value: Pearson’s Chi-Square test was used to compare the prevalence of the single somatic symptoms between younger and older patients. Bold numbers indicate that the difference between the age groups is statistically significant (p < 0.05).

* PRIME-MD = Primary Care Evaluation of Mental Disorders.

### Course of somatic symptoms

A successive decrease in the mean number of somatic symptoms was seen for the total group at each follow-up; baseline 5.3 (SD 2.4), three months 4.0 (SD 2.6) (p < 0.0005), six months 3.7 (SD 2.5) (p = 0.006), 12 months 3.3 (SD 2.3) (p = 0.012) and 18 months 3.1 (SD 2.5) (p = 0.028). A similar pattern was seen for both sexes, but the decrease between 3 and 6 months did not reach statistical significance in either women or men (data not shown). Also, the decrease in the number of symptoms among women between 12 (mean 3.5; SD 2.3) and 18 months (mean 3.3; SD 2.6) was not significant (p = 0.264). At the final follow-up, 18 months after onset of treatment, men reported a mean of 2.5 (SD 2.4) somatic symptoms and this was the only time point showing a statistically significant sex difference, with women reporting a mean of 3.3 (SD 2.6) (p = 0.024). There were no clear differences in this respect when comparing younger and older patients.

**Figure 1:**
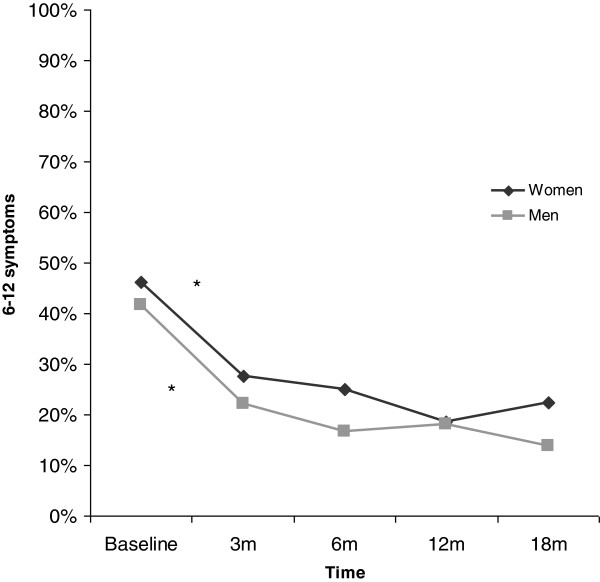
**Proportion of women and men reporting six symptoms or more at baseline and at follow-ups after 3, 6, 12 and 18 months.** *Indicates that the difference between two time points is statistically significant (p < 0.05).

The proportion of patients reporting six symptoms or more declined from baseline (45%) to follow-up at 18 months (20%) (p < 0.0005). Most of this reduction in symptoms occurred during the first three months. The pattern was similar for women and men (Figure 1), as well as for younger and older patients.

### The course of single symptom

**Figure 2:**
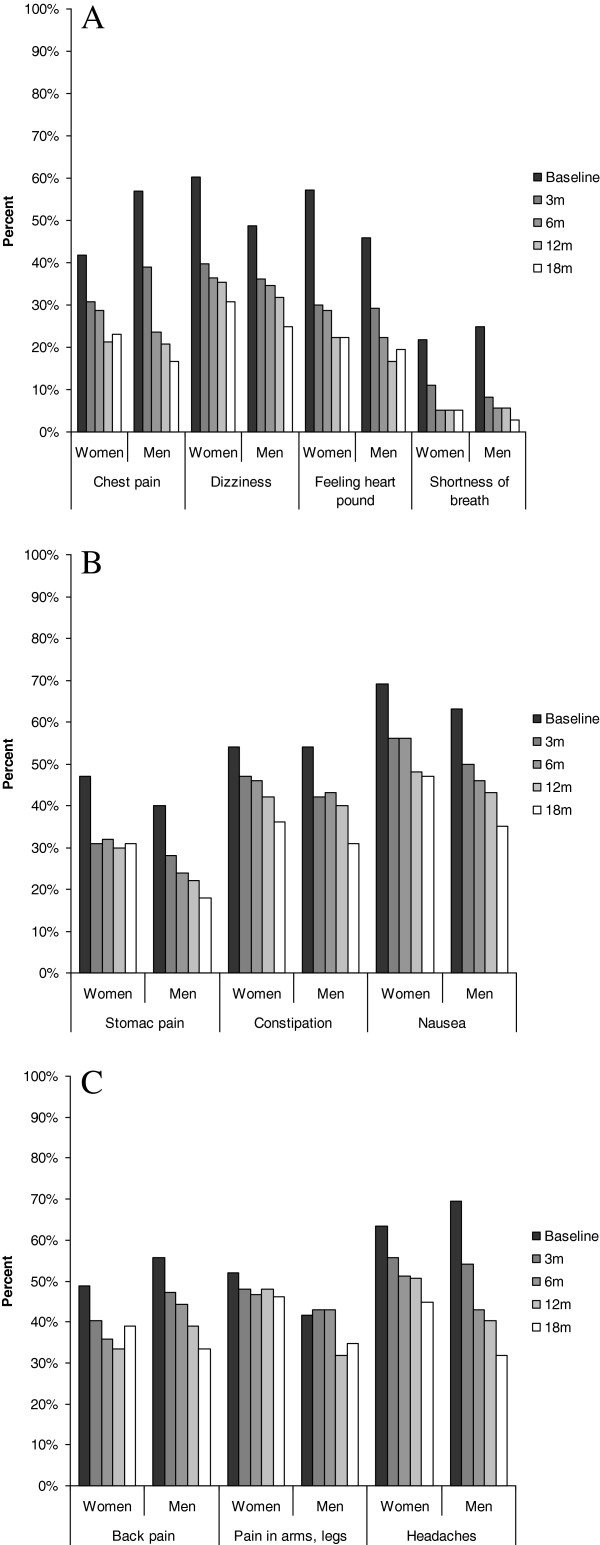
**Course of somatic symptoms. A**. Course of symptoms from the chest and dizziness during 18 months follow-up in male and female patients with stress-related exhaustion. The only significant difference seen was that chest pain was more common among men (57%) than women (42%) at baseline (p = 0.032). **B**. Course of gastrointestinal symptoms during 18 months of follow-up in male and female patients with stress-related exhaustion. The only significant difference seen was that stomach pain was more frequently reported among women (31%) than men (18%) (p = 0.035). **C**. Course of pain-related symptoms during 18 months in male and female patients. At 12 months pain in arms, legs or joints was more common among women (48%) than men (32%) (p = 0.022).

The prevalence of all 12 somatic symptoms assessed in this study decreased between baseline and the final follow-up at 18 months, except for pain in arms, legs or joints (p = 0.401), and fainting spells (due to the small number of cases). Thus, neither in women nor in men was there a significant decline in the prevalence of pain in arms, legs or joints. Similarly, sexual problems were more often reported by men at baseline (21%) than by women (10%), but at follow-up only 4% of the men still had such problems (p < 0.0005) with no difference between the sexes. The course of somatic symptoms was similar among younger and older patients, with one exception showing that pain in arms, legs or joints was significantly more common in older patients (58%) compared to younger (33%) (p < 0.0005) at baseline. This difference between older and younger was present during the course of all measurement points (data not shown). The development of the respective symptoms over 18 months is shown in Figure 2a, b and c.

### Somatic symptom in relation to symptoms of exhaustion, depression and anxiety

Patients scoring above the median of 5.45 on the burnout scale SMBQ *(n = 105)* reported a significantly higher number of somatic symptoms (mean 6.1; SD 2.5) than patients with lower scores *(n = 115)* (mean 4.5; SD 2.1) (p < 0.0005). Similarly, when the patients were split into two groups with cut-off 11 or more on the HAD scale, patients scoring above the cut-off for symptoms of depression (n = 74) reported more somatic symptoms (mean 6.0; SD 2.5) than those scoring below (n = 140) (mean 4.9; SD 2.3) (p = 0.002). High anxiety levels *(n = 132)* was also associated with a higher number of somatic symptoms (5.9; SD 2.5) when compared with patients with lower scores *(n = 81),* (mean 4.4; SD 2.1) (p < 0.0005).

### Predictors of recovery

All patients with complete data who reported six or more symptoms at baseline (n = 102) were included in the analysis regarding possible predictors (level of education, marital status, symptom duration before seeking medical care, co-morbidity of depression and/or anxiety, use of ADs, sick leave and physical activity) of having less than six symptoms at follow-up. None of the variables tested were found to predict recovery at any of the follow-ups. Data from the 12-month follow-up are shown in Table 5 and similar results were found for other time points.

**Table 5:** Results from Cox’s regression analyses of possible predictors of reporting less than six somatic symptoms at 12-month follow-up in patients with Exhaustion Disorder who reported six symptoms or more at baseline (N = 102)

	**N**	**RR (95% CI)**
Sex		
- Men	30	1
- Women	72	1.01 (0.59-1.72)
Age		
- 18-39	37	1
- 40-64	65	0.85 (0.52-1.40)
Co-morbid depression		
- No	18	1
- Yes	84	0.66 (0.37-1.15)
Co-morbid anxiety disorder		
- No	12	1
- Yes	90	0.73 (0.37-1.44)
Marital status		
- Married	76	1
- Single or other	26	0.80 (0.44-1.45)
Symptom duration		
- less than a year	37	1
- one year or more	61	0.74 (0.45-1.22)
Sick leave		
- No	29	1
- Part time	16	1.39 (0.67-2.85)
- Full time	57	1.05 (0.59-1.87)
Education, college or more		
- Yes	60	1
- No	28	1.36 (0.79-2.36)
Physical activity		
- Sedentary lifestyle	27	1
- Light physical activity	50	1.05 (0.58-1.89)
- Moderate or intense physical activity	22	0.87 (0.41-1.81)

Risk ratio (RR) and 95 % confidence interval (CI).

## Discussion

The main findings from this study are that patients seeking medical care mainly for stress-related exhaustion, also report a numerous different somatic complaints. Thus, almost half of the participants reported six somatic symptoms or more at the first consultation. Concomitantly to that the patients are receiving treatment for the exhaustion, most of the somatic symptoms decline. Neither number of somatic symptoms or changes in number symptoms reported over time differ between men and women or between younger and older patients.

It is well known from the literature that somatic symptoms are closely related to mental symptoms both in general populations and in primary care populations [15,45]. This study has focused on a specific patient population that seeks medical care mainly for stress-related mental symptoms and it is thus expected that the symptoms that were reported by the patients in this study are strongly related to psychosocial stress [1]. The interesting finding is, however, that female and male patients do not differ regarding the burden of somatic symptoms, which resembles the findings from our previous study measuring mental symptoms [6]. Thus, these results differ from what is usually seen in primary care populations, i.e. that women report a heavier burden of symptoms. The PRIME-MD 1000 study in primary care showed that women more commonly reported as many as 10 out of 13 somatic symptoms to be prevalent during the preceding months [29]. A Swedish study in primary care showed that women report more somatic symptoms during the preceding 3 months than men [46]. Furthermore, the HUNT-II study covering all inhabitants in one region in Norway more than 20 years of age showed that women compared with men report more somatic symptoms during the preceding year [36]. One may speculate that sex differences commonly seen with regard to both mental and somatic symptoms vanish when the health problems reach such a severe level as is the case for the patients included in this study.

Concerning specific symptoms the only difference seen between the sexes in the present study was a higher prevalence of chest pain and pain or problems during sexual intercourse among males. In line with this present study stress at work was associated with an almost fourfold increase in the risk for unexplained chest pain among men, whereas this was not seen among women [47], and burnout has previously been shown to risk factor for cardiovascular health problems among men while the evidence is unclear in women [24,48]. This symptom does decline with time, concomitantly as the patients are being treated to their stress problems, and no difference was seen between the sexes during the last follow-up 18 months later*.* This supports the hypothesis that the origin of the chest pain in this group of patients is stress-related and that men report this symptom more frequently than women when experiencing stress. In line with our finding, pain or other problems during sexual intercourse was the only symptom noted to be more common among men than women in a patient population seeking primary care [29]. This is a rare symptom but should be taken into consideration since it was reported by more than one fifth of the men and most probably reduces quality of life substantially. Fortunately, it seems that the treatment offered at the clinic succeeded in influencing this clinical problem since only four per cent of the male patients reported this to be a problem at 18-month follow-up. We do not, however, know if they had been seeking treatment elsewhere for this problem, which is a limitation when drawing a conclusion regarding the reason for symptom reduction.

There was no difference in the average number of somatic symptoms between younger and older patients: earlier studies on primary care populations showed divergent results for somatic symptoms and association with age [46,49]. Only one symptom was found to differ between the groups, showing that older patient more commonly report pain in arms legs and joints. It has been shown that the prevalence of somatic symptoms increases with age in general population [36], but this does not seem to be the case in patient with stress-related exhaustion. One may speculate that a similar explanation could be offered as in the discussion about sex differences above, i.e. potential differences regarding both mental and somatic symptoms vanish when the health problems reach such a severe level as is the case for the patients included in this study. Thus, even patients still only in their twenties report a similar burden of symptoms as patients in their sixties and consequently the burden of somatic symptoms even among young patients with exhaustion might be expected to be high. Pain in arms legs and joints in older has to be specially considered to offer adequate treatment.

The most prevalent symptoms in this study are commonly regarded as being stress-related and a recent review confirms that this is the case for e.g. gastrointestinal problems, dizziness, headaches and back pain [1]. In fact, many of the patients referred to the clinic report during the clinical interview that they had earlier been seeking medical help for one or more of their somatic symptoms without recognition of the underlying stress exposure and associated mental health problems. In primary care it is common that patients seek help for a single symptom, even though they are in fact suffering from several other mental and/or somatic symptoms [49]. This indicates that even among patients seeking health care mainly for one symptom of the kind discussed here, it is important to check for multiple stress-related symptoms, thereby increasing the possibility of preventing the development of more severe stress-related conditions such as exhaustion.

An improvement regarding most symptoms with time, similar for both sexes and age groups was shown. No specific treatment was offered for the single somatic symptoms. All patients received individualised MMT mainly including measures to reduce stress exposure. As this study is not designed as an intervention study, we cannot conclude whether the MMT treatment offered at the clinic is responsible for the improvement in somatic symptom. We can thus only cautiously conclude that the treatment offered seems to offer general stress reduction and probable effects of stress-related symptoms in general. A substantial improvement the first three months after the initial visit at the clinic was shown. This early reduction in symptoms could be an effect of the initial consultation offering support and care. This brings about relief of worries, as do the offers of sick leave or various rehabilitation activities. The general aim of the treatment programme at the clinic is reduce both the stress exposure and the perceived stress levels and it is likely that this is having an effect on both mental and somatic symptoms.

Even though the prevalence of most symptoms gradually decreases, one fifth of the patients were still reporting six symptoms or more at the 18-month follow-up. This reduction of symptoms seems, however, to be greater for patients with ED than for patients with somatisation, as about 18 per cent of the patients in this study reported six symptoms or more after 12 months compared to around half of the participants in a study of female patients with somatisation [50]. We can cautiously conclude that patients with ED seem to differ from patients with somatisation and the core problem for the ED-patients is the mental exhaustion. The somatic symptoms that co-exist with that exhaustion seem to be mainly stress-related which gradually decrease with time as the patient is being treated for the stress problems.

The only exception in regard to reduction of symptoms during MMT treatment is pain in arms, legs and joints, reported by around half of the patients, both men and women. This problem is more pronounced in older patients with stress-related exhaustion. The prevalence does not change significantly over time and this is one of the most common symptoms reported by both men and women during the 18-month follow-up. Thus, such localised pain problems are not relieved concomitantly as the patients are being treated for the stress problems. This is an interesting finding as multimodal treatment programme directed towards pain has been shown to be successful, at least in patients seeking medical care predominantly for musculoskeletal pain problems [51]. Thus, it seems that the MMT used in our clinic, tailored to relieve the mental symptoms but also having a positive effect on most of the somatic symptoms should be supplemented with additional modalities in cases with pronounced localised pain. Such an approach should be considered early in the treatment of this type of patient. Our finding regarding lack of improvement in musculoskeletal symptoms in patients with mental health problems is not unique as several previous studies have reported similar results. In a review 2003, it was shown that patients with both depression and pain experienced longer duration of pain and poorer overall response to treatment than patients experiencing pain without co-morbid depression [52]. A study of patients with depression randomly assigned to care as usual or to one of two collaborative management programmes showed that patients randomised to the intervention group reported significantly fewer somatic symptoms at follow-up except for current pain symptoms [53]. The majority of the patients in this study suffered from co-morbid depression at baseline which might have contributed to the large proportion still reporting pain at the final follow-up. Additional analysis also showed that this group scored significantly higher on symptoms of depression, but not on symptoms of burnout, compared to those without pain, which confirms previous research showing that patients reporting both depression and pain symptoms experience a longer duration of pain problems [52].

One of our aims was to explore if reduction in somatic symptoms could be predicted by factors that could be of importance for the course of illness. Interestingly, co-morbid depression or anxiety, sick leave and physical activity did not predict the decrease of somatic symptoms in patients with multiple symptoms at baseline in our study. This is different to what has previously been shown regarding the development of persistent somatisation, i.e. that anxiety, depression, marked impairment, and older age are predictors, according to a review by Creed and Barsky [54]. Symptom duration was the only factor found to predict recovery from mental symptoms in our previous study of the same patient group [8].

The course of improvement of somatic symptoms in patient with stress-related mental exhaustion as a core feature seems to be more related to the stress reduction than to factors that otherwise might predict recovery, e.g. in patients with somatisation, when the somatic symptoms are the core feature.

There are several limitations that should be mentioned regarding this study. Firstly, this is not an intervention study and the purpose is not to relate treatment to symptoms as all patients received similar treatment. We can thus not make a firm conclusions that the treatment offered at the clinic is solely responsible for the reduction in somatic symptoms reported by the patients. Another important limitation is that the referral procedure has resulted in a selection of a patient population that is quite highly educated with presumably high health literacy and knowledge of the possibilities and usability of the health care system. Thus, the results are not easy to generalise to other groups. To ensure that the study group is reasonably homogeneous with regard to duration of symptoms and sick leave, only data from patients reporting sick leave shorter than six months was analysed in this study. The treatments that had been undertaken prior to the consultation at the stress clinic varied somewhat, especially regarding AD medication. We did not find any difference between patients with and without ADs in the recovery, either with regard to mental or somatic symptoms in this study. Another limitation is that the content of the MMT at the stress clinic could differ somewhat between the patients due to individual needs, but we have no reason to believe that this constitutes any major bias in this study when comparing different groups i.e. men and women or younger and older patients. It should also be mentioned that the somatic symptoms were measured using a symptom checklist and no measure of severity was performed. A symptom checklist like the PRIME MD that simply inquires about the presence or absence of a symptom may overestimate clinically relevant symptoms. Finally, questions could be raised with regard to common method variance as a limitation. Physical symptoms are included in the ED diagnosis as one of six items under criterion C. However, only four out of six items on C have to be answered positive for an ED diagnosis. Thus, reporting somatic symptoms are possible but not obligatory in order to be diagnosed with ED. Since patients with stress-related exhaustion without any such symptoms still can meet the diagnostic criteria it does not seem plausible that the prevalences of somatic symptoms are overestimated.

The major strength of this study is that it comprises a large number of patients from the same clinic, diagnosed and treated by only three different senior physicians. A long-term follow-up of 18 months is studied. To our knowledge this is the first study presenting the prevalence and course of somatic symptoms in a clinical patient population with mental exhaustion/burnout.

## Conclusion

In conclusion, this study provides important knowledge regarding somatic symptoms in patients with stress-related exhaustion. The prevalence and course of symptoms were not related to sex or age. Somatic symptoms were strongly related to mental symptoms at baseline but, interestingly, co-morbid depression or anxiety did not predict recovery of somatic symptoms. Most of the symptoms seem to be stress-related and concomitantly with that the patients are being treated for the stress-related exhaustion, reduced prevalence is seen for almost all somatic symptoms. One important exception is pain in the arms, legs and joints which were highly prevalent still after 18 months. Thus, special attention should be paid to such symptoms especially in older patients. This study points out the importance of acknowledging the large number of somatic symptoms often present in these patients, without overlooking the core problems of exhaustion and co-morbid mental health symptoms such as depression and anxiety.

## Competing interests

The authors declare that they have no competing interests.

## Authors’ contributions

KG has made substantial contributions to the data collection, concept, design and data analysis, and for writing the manuscript. She has also clinically examined and treated at least half of the patient group for 18 months. GA has made substantial contributions to the concept, design and data analysis, and revised the manuscript critically. IJ has made substantial contributions to the concept, design and data analysis, and revised the manuscript critically. All authors read and approved the final manuscript.

## Pre-publication history

The pre-publication history for this paper can be accessed here:

http://www.biomedcentral.com/1471-244X/14/118/prepub
